# Impact of overactive bladder on quality of life and resource use: results from Korean Burden of Incontinence Study (KOBIS)

**DOI:** 10.1186/s12955-015-0274-9

**Published:** 2015-06-26

**Authors:** Kyu Sung Lee, Myung Soo Choo, Ju Tae Seo, Seung June Oh, Hyeong Gon Kim, Kwong Ng, Kyung Jin Lee, Jonathan T. Tan, Joon Chul Kim

**Affiliations:** Samsung Medical Center, Sungkyunkwan University School of Medicine, 81 Irwon-Ro, Gangnam-Gu, Seoul South Korea; Asan Medical Center, University of Ulsan College of Medicine, 88 Olympic-Ro, 43-Gil, Songpa-Gu, Seoul South Korea; Cheil General Hospital, Dankoo University College of Medicine, 1-19 Mukjeong-Dong, Jung-Gu, Seoul South Korea; Seoul National University Hospital, 101 Daehak-Ro, Jongno-Gu, Seoul South Korea; Konkuk University School of Medicine, Konkuk University Medical Center, Gwangjin-Gu, Seoul South Korea; Allergan APAC, Pasir Panjang Road, MBC, West Building #09-25, Singapore, 117439 Singapore; Allergan Korea, GT Tower 14F, 1317-23 Seocho-dong, Seocho-gu Seoul; Bucheon St.Mary’s Hospital, 2 Sosa-Dong, Wonmi-Gu, Gyeonggido South Korea

**Keywords:** Overactive urinary bladder, Urinary incontinence, Quality of life, Cost of illness

## Abstract

**Background:**

To evaluate the impact of overactive bladder (OAB) on quality of life (QOL), resource use and productivity loss in patients recruited from six hospitals in Korea.

**Methods:**

This cross-sectional survey recruited 625 OAB patients between July to December 2013. Patients were categorised into four groups based on the average number of urinary incontinence (UI) episodes over the past three days (0, 1, 2–3 and ≥4 UI/day). QOL was measured using the Incontinence-Specific Quality of Life Instrument (I-QOL), the Overactive Bladder Questionnaire (OAB-q), and a generic health-related utility instrument (EQ-5D). Information on hospital and clinic visit frequency, and continence pads use were also collected. Work productivity was assessed using the Work Productivity and Activity Impairment (WPAI) questionnaire. Between group differences were assessed using ANOVA. Multivariable regression analyses were performed to examine the independent effects of OAB symptoms on QOL.

**Results:**

Severity of UI showed a significant linear relationship with QOL, with clinically meaningful differences between each UI severity category. Compared to the dry category, patients in the most severe category (≥4 UI/day) had significantly lower I-QOL scores (69.8 vs 42.6; *p* < 0.0001), greater symptom bother on the OAB-q (30.4 vs 64.6; *p* < 0.0001), and poorer EQ-5D utility (0.848 vs 0.742; *p* < 0.001). Multivariable analyses showed that UI severity, frequency, urgency, and nocturia are independently associated with poorer QOL. Incontinence severity is also significantly associated with cost of incontinence pads (*p* < 0.0001), and a greater interference with work and regular activities (*p* = 0.001), however, no significant difference in hospital and clinic visits were observed.

**Conclusion:**

Severity of UI is a key contributor to the disease burden of OAB in Korean patients, even after taking into account the impact of other symptoms associated with OAB.

**Electronic supplementary material:**

The online version of this article (doi:10.1186/s12955-015-0274-9) contains supplementary material, which is available to authorized users.

## Introduction

Overactive bladder (OAB) is characterized by symptoms of urgency with or without urinary incontinence (UI), and usually with urinary frequency and nocturia [1, 2]. The study by Agarwal *et al.* (2014) [3] assessed lower urinary tract symptoms and found that while urinary urgency is a common troubling symptom, UI was the most bothersome symptom to the individual. Urinary incontinence has a multi-dimensional impact on quality of life (QOL), affecting the physical, social, psychological, and domestic aspects of patients’ lives [4–6]. The ability to control of one’s elimination functions is a highly valued health outcome. Consequently, the lack of such control (ie, incontinence) is associated with a higher risk of psychological distress [7, 8].

In addition, OAB results in substantial direct costs relating to the management of UI, and an increased likelihood of co-morbidities that include falls, urinary tract infections, depression, and sexual health problems [9–11]. The burden of OAB is also attributed to decreased productivity and lost wages [12, 13]. The recent International Burden of Incontinence Study (IBIS) [14] had shown healthcare resource utilization (eg, clinic visits) in western countries increased with UI severity.

With the prevalence of UI in Asian countries ranging from 2.6 to >30 %, depending on the type of incontinence and age of population [15–17], UI represents a significant societal and financial burden [2, 18]. In Korea, the prevalence of UI reported in the 2007–09 Korean National Health Survey was 7.9 % [19]. The 2005 Korean EPIC study [20] reported an OAB prevalence of 12.2 %, while OAB with UI was 5.1 %. Accordingly, the present study aims to assess the relationships between QOL, resource use, and productivity loss with UI severity in a Korean population suffering from OAB.

## Methods

### Study design and patients

This was a cross-sectional, clinic-based observational survey with individuals recruited through six hospital urology clinics in Korea between July to December 2013.

Patients enrolled in the survey were aged 18 years and above and diagnosed with idiopathic OAB by a urologist. Participants were required to have OAB with at least one episode of urinary incontinence in the past 12 months. To exclude neurological disorders that could cause similar symptoms with OAB, participants with a diagnosis or history of a neurological condition (eg, traumatic brain injury, spinal cord injury, multiple sclerosis) were ineligible for the study. Other exclusion criteria were predominance of stress UI (where >50 % of UI episodes are provoked by physical exertion), history of bladder or prostate cancer, or other bladder related conditions (eg, interstitial cystitis, bladder stone, bladder reconstructive surgery).

Participants were asked to recall the number of incontinence episodes in the past 3 days, which was used to calculate the average number of UI/day for appropriate group allocation (dry, 1 UI/day, 2–3 UI/day, ≥4 UI/day).

All participants provided written consent. Participants completed the 30-minute on-line questionnaire at the clinics and were compensated (KRW 30,000, approximately US$28) for their time. The study was approved by the respective institutional ethics review boards in the six hospitals.

### Measures

The Incontinence Quality of Life (I-QOL) questionnaire is a commonly used and well-validated instrument [21, 22] with a validated Korean version [23]. The I-QOL consists of 22 items, each with five ordinal response levels. The 22 items are grouped into 3 subscales: Avoidance and Limiting Behaviour, Psychosocial Impacts, and Social Embarrassment. The summary and subscale scores showed excellent reliability (Cronbach’s alpha 0.91-0.96), reproducibility (intra-class correlation coefficient 0.72-0.97), and validity demonstrated by significant correlation with independent measures of incontinence severity [24]. The I-QOL has a 0–100 point scale where a lower score indicates greater impact of incontinence on QOL. A change of ≥4 in the I-QOL total score is regarded to be the minimal clinically important difference (MCID) [25].

The Overactive Bladder questionnaire (OAB-q) symptom is a validated OAB-specific QOL instrument [26], with a validated Korea version [27]. The OAB-q symptom subscale consisting of eight items, each with six ordinal response levels. The total and subscales showed excellent reliability (Cronbach’s alpha 0.77-0.95), reproducibility (intra-class correlation coefficient 0.55-0.77), and validity demonstrated by significant differences between the scores for OAB patients and controls [27]. All scale scores are transformed to a 100 point scale with higher scores indicating greater symptom severity. The MCID for OAB-q symptom subscale has been identified to be ≥10 [28, 29].

The EuroQOL (EQ-5D) 5 L is a generic health related utility instrument with five dimensions (mobility, self-care, usual activities, pain/discomfort, and anxiety/depression) and general health status. The health states are converted into a weighted index that ranges from 0 to 1, with 1 being full health and 0 being death. The EQ-5D has been validated for use in Korea [30], however, as no Korean value datasets are available, the UK valuation set is used to derive utility values. The MCID for utility instruments is considered to be ≥0.03 [31, 32].

### Resource use and work productivity

Healthcare utilization data collected include health practitioner visits, and inpatient and outpatient hospitalizations. Weekly usage of incontinence pads by capacity (eg, thin, medium, heavy, overnight, diaper) was also collected. Average cost of pads estimated using prices from the largest online wholesale retailer in Korea (www.emart.com). Work productivity was assessed using the validated Work Productivity and Activity Impairment (WPAI) specific health problem questionnaire [33]. The WPAI consist of 6 items, which measure the impact of incontinence on working hours lost and the degree of interference with productivity and regular activities. Caregiver burden was measured as the number of hours of caregiver aid provided by paid and non-paid helpers each week.

### Statistical analyses

All participants who met the entry criteria and completed the survey were included in the analyses. The survey population was categorised into four groups based on UI severity (ie, 0, 1, 2–3 and ≥4/day), determined by the average number of UI episodes per day in the preceding three days. Based on the ability to detect a clinically meaningful difference of 5 points in I-QOL scores between each group (SD = 15) [34] with 80 % power (two-sided α = 0.05), the minimal sample size of study was estimated to be 600.

Statistical analyses were performed using STATA (12.0, College station, TX). Between group differences were assessed using ANOVA and linear regression for continuous outcomes, and logistic regression for dichotomous outcomes. Multivariable regression was used to adjust for co-variates including age, gender, income, education, and OAB treatment status where appropriate. Pairwise correlations between variables were examined to assess for collinearity. Taking into account the presence of multiple comparisons, a significance threshold of *p* < 0.01 was utilised, balancing the risk of type I and II errors.

## Results

Six hundred tewenty-five participants were screened and deemed eligible with all participants completing the survey. The socio-demographic and clinical characteristics are shown in Table 1. The mean age was 63.5 years; 68 % were female of whom 86.7 % post-menopausal. Comparison across the UI severity groups showed significant association between increasing UI severity with proportion of women (*p* < 0.001), and with education level (*p* = 0.009). The overall mean UI episodes/day was 2.02. As expected, the incidence of key OAB symptoms including urinary urgency (*p* < 0.001), frequency (*p* = 0.002) and nocturia (*p* = 0.006), were also positively associated with UI severity. The prevalence of co-morbidities in the past six months were generally low and did not vary significantly across UI severity groups except for skin infections due to UI (*p* = 0.002) and presence of high blood pressure (*p* = 0.005) (Additional file 1: Table S1).

**Table 1:** Socio-demographics and disease characteristics of patients

	All patients	Incontinence episode per day				
		0	1	2–3	≥4	*p*-value*
N	625	162	165	177	121	
Age (SD)	63.5 (12.0)	62.3 (12.2)	63.2 (13.3)	63.9 (11.6)	65.0 (10.3)	0.037
Female, %	422 (67.6)	93 (57.8)	100 (60.6)	131 (74.0)	98 (81.0)	<0.001
• % Post-menopausal	86.7	82.8	87.0	89.3	86.7	0.983
% retired	26.9	29.0	33.3	22.0	22.3	0.195
Education level, %						
• Elementary/middle school	36.2	27.8	30.9	39.0	50.4	0.009
• High school graduate	36.4	36.4	38.2	39.0	30.6	0.690
• University graduate	27.4	35.8	30.9	22.0	19.0	0.020
Household income, %						
• 0 – 50,000,000 KRW	74.8	69.3	77.8	78.4	79.1	0.341
• 50,000,001 – 100,000,000 KRW	16.3	19.7	14.8	11.9	20.5	0.793
• > 100,000,000 KRW	8.9	10.9	7.4	9.7	6.4	0.559
Health insurance coverage (%)						
• National Health Insurance	71.5	71.6	69.7	70.1	76.0	0.909
• National Health Insurance and Private Insurance	26.3	27.2	27.9	27.6	20.7	0.515
• Public Assistance	2.2	1.2	2.4	2.3	3.3	0.110
OAB characteristics						
Urinary incontinence, episodes/day (SD)	2.02 (2.31)	0 (0)	1 (0)	2.43 (0.50)	5.54 (2.80)	<0.001
Daytime urgency, episodes/day (SD)	3.95 (3.71)	2.78 (2.65)	3.19 (2.60)	3.90 (2.77)	6.64 (5.64)	<0.001
Daytime micturition, episodes/day (SD)	8.94 (4.29)	8.70 (6.27)	8.75 (3.24)	8.84 (3.16)	9.67 (3.63)	0.134
% with urinary frequency^a^	43.4	36.4	41.2	44.6	53.7	0.002
Nocturia, episodes/day (SD)	2.39 (2.23)	1.95 (1.38)	2.44 (3.00)	2.33 (1.70)	3.00 (2.50)	0.006
Use of anti-cholinergic^b^ medication, n (%)						
• Currently	41.6	50.0	45.5	39.0	28.9	<0.001
• In the past	8.3	4.3	6.1	9.0	15.7	0.001
• Never	50.1	45.7	48.5	52.0	55.4	0.019

*Comparison across severity groups conducted using linear or logistic regression for continuous and dichotomous outcomes, respectively. Where appropriate, analysis adjusted for age and gender

^a^Urinary frequency defined as ≥9 micturition episodes per day

^b^Anti-cholinergic medications include Tolterodine, Propiverine, Oxybuytnin, Trospium, Fesoterodine, Solifenacin, Flavoxate, and Imipramine

The current use of anti-cholinergics was negatively associated with UI severity (Table 1). The rate of current anti-cholinergic use was significantly greater in the dry group versus the most severe UI group (50 % vs 29 %, *p* < 0.001). Conversely, the rate of previous anti-cholinergic use was positively correlated with UI severity (*p* = 0.001). History of other OAB treatments were generally similar across UI severity groups (Additional file 1: Table S2). The majority of participants had not received bladder training (70.6 %), acupuncture (95.9 %), clean intermittent catheterisation (97.5 %), indwelling catheterisation (99.2 %), herbal therapy (93.9 %), while 96.6 % of patients had never undergone any medical procedures for the treatment of OAB (eg, botulinum toxin injections, electrical stimulation, bladder denervation, detrusor myomectomy). The willingness of OAB patients to try such medical procedures to improve their urinary symptoms was positively associated with UI severity (Table 4, *p* < 0.001).

**Fig. 1:**
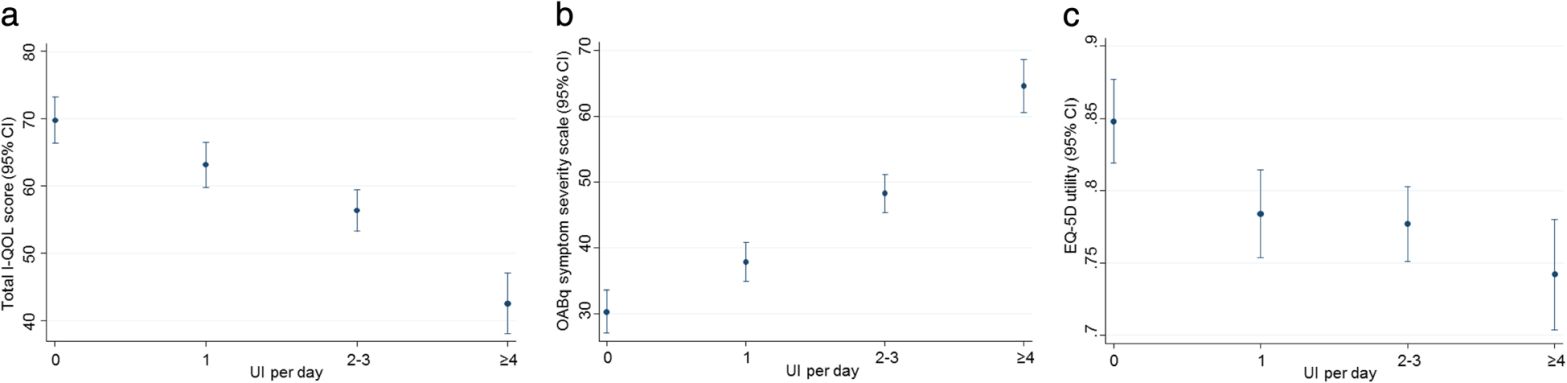
Urinary incontinence severity is significantly associated (*p* < 0.001) with QOL as measured by (**a**) I-QOL total score, (**b**) OAB-q symptom severity score, and (**c**) EQ-5D utility

The impact of UI on QOL was assessed using two incontinence-specific instruments (I-QOL and OAB-q), and a generic health-related utility instrument (EQ-5D). As shown in Fig. 1, increasing UI severity was consistently associated with a significant reduction in QOL as measured by all three instruments (*p* < 0.001). The I-QOL total score was highest in the dry group (69.8), compared to 42.6 in the most severe group (<0.0001), indicating a score difference of at least 27 points between the two groups. There was a minimum difference of 5 points in I-QOL score observed between each UI severity group. This indicates that even 1 UI episode per day has a clinically significant impact on the QOL of OAB patients (Table 2). Similarly, UI severity was significantly associated (*p* < 0.0001) with poorer score in the three I-QOL subscales for avoidance and behaviour limiting, psychosocial impact, and social embarrassment. Results for the OAB-q severity subscale showed greater bothersome symptoms with increasing UI severity (30.4 in dry group compared to 64.6 in most severe group; *p* < 0.0001). The EQ-5D utility scores also showed a significant association with UI severity (*p* = 0.0006). OAB patients in the dry group had the highest average utility score compared to patients in the most severe groups (0.848 vs 0.742; *p* < 0.001). Importantly, the differences observed between UI severity groups exceeded the MCID for all three QOL instruments.

**Table 2:** Impact of urinary incontinence severity on quality of life

	All patients	Incontinence episode per day				
		0	1	2–3	≥4	*p*-value*
N	625	162	165	177	121	
I-QOL Total score (SD)	59.0 (24.3)	69.8 (22.1)	63.2 (22)	56.4 (21.1)	42.6 (25)	<0.0001
• Avoidance and limiting behaviour subscale	57.9 (23.5)	68.6 (21.8)	61.8 (21.6)	55.3 (20.2)	42.0 (23.5)	<0.0001
• Psychosocial impact subscale	64.6 (26.4)	73.7 (24.5)	68.2 (23.8)	63.0 (23.9)	50.1 (29.3)	<0.0001
• Social embarrassment subscale	50.7 (27.8)	64.7 (23.8)	56.6 (25.5)	46.4 (24.9)	30.1 (26.4)	<0.0001
OAB-q – Symptom severity scale (SD)	44.1 (23.9)	30.4 (21.4)	37.9 (19.5)	48.3 (19.7)	64.6 (22.7)	<0.0001
EQ-5D utility score (SD)	0.791 (0.199)	0.848 (0.188)	0.784 (0.198)	0.777 (0.176)	0.742 (0.214)	0.0006

*Test for association between incontinence episodes and QOL measure. Adjusted for age and gender

In recognition of OAB as a symptom complex, the two incontinence-specific instruments were further analysed using multivariable regression to examine the relationship between UI and QOL, while accounting for other key OAB symptoms (urinary frequency, urgency and nocturia), age, gender, and OAB treatment status. As shown in Table 3, UI/day, frequency, urgency, and nocturia were all independently associated with poorer QOL, with each factor contributing to a clinically significant change in the I-QOL total score, as shown by negative coefficients of >4 in the multivariable model (*p* < 0.0001; adjusted R^2^ = 0.382). A similar pattern was observed using the OAB-q symptom scale, with the presence of two or more symptoms resulting in clinically meaningful change in the symptoms severity of OAB to patients. This was shown by a total coefficient score of >10 when the individual coefficients of respective symptoms were combined (*p* < 0.001; adjusted R^2^ = 0.543). However, UI did not remain a significant factor in multivariable analysis of EQ-5D (*p* = 0.174).

**Table 3:** Multivariable regression to assess impact of OAB symptoms on QOL

Variables included in model	Coefficient	95 % CI	*p*-value
Multivariable model for IQOL total score (Adjusted R^2^ = 0.394)			
UI category	−5.09	−6.62, −3.55	<0.0001
Urinary frequency	−5.08	−8.36, −1.79	0.0025
Urinary urgency	−4.29	−5.54, −3.03	<0.0001
Nocturia	−5.00	−6.31, −3.69	<0.0001
Receiving treatment for OAB	−5.99	−9.23, −2.74	<0.0001
Age	0.29	0.15, 0.41	<0.0001
Gender	−8.60	−11.85, −5.35	<0.0001
Multivariable model for OAB−q – Symptom severity score (Adjusted R^2^ = 0.543)			
UI category	7.08	5.77, 8.39	<0.0001
Urinary frequency	6.38	3.56, 9.20	0.0029
Urinary urgency	5.58	4.51, 6.66	<0.0001
Nocturia	6.00	4.88, 7.12	<0.0001
Age	−0.15	−0.26, −0.04	0.007
Gender	−0.16	−2.94, 2.62	0.912
Multivariable model for EQ−5D utility (Adjusted R^2^ = 0.121)			
UI category	−0.01	−0.03, 0.005	0.174
Urinary frequency	−0.03	−0.06, 0.005	0.098
Urinary urgency	−0.02	−0.03, −0.008	0.001
Nocturia	−0.02	−0.04, −0.01	0.001
Age	−0.0005	−0.002, 0.001	0.457
Gender	−0.08	−0.11, −0.05	<0.0001

All variables included in the multivariable models for each of the QOL measures are shown above. Income and education did not show statistical significance and were not included in the models. OAB treatment status was only statistically significant for I−QOL

The association between UI severity and resource use are examined in Table 4. While there appeared to be a trend of greater visits to hospital, clinic, and emergency care with increased UI severity, the differences were not statistically significant. However, UI severity was associated with increased weekly cost of incontinence pads, greater impact on work productivity, and a greater interference with regular activities (*p* ≤ 0.001).

**Table 4:** Healthcare resource utilization and economic impact of incontinence

	All patients	Incontinence episode per day				
		0	1	2–3	≥4	*p*-value
N	625	162	165	177	121	
Heath service utilization, mean number of visits over 6 month (SD)						
Outpatient	1.42 (2.28)	1.30 (2.34)	1.35 (2.29)	1.48 (1.76)	1.61 (2.79)	0.484
• Hospital	1.01 (1.53)	0.80 (1.38)	0.95 (1.63)	1.16 (1.44)	1.13 (1.69)	0.073
• Clinic	0.35 (1.30)	0.48 (1.67)	0.34 (1.40)	0.24 (0.68)	0.35 (1.29)	0.152
• Oriental clinic	0.07 (0.66)	0.03 (0.25)	0.06 (0.55)	0.07 (0.43)	0.13 (1.21)	0.289
Inpatient	0.05 (0.26)	0.06 (0.28)	0.06 (0.23)	0.03 (0.17)	0.09 (0.37)	0.736
• Hospital	0.05(0.25)	0.05 (0.24)	0.05 (0.22)	0.03 (0.17)	0.09 (0.37)	0.537
• Clinic	0.003 (0.06)	0.006 (0.08)	0.006 (0.08)	0	0	0.247
Emergency care	0.014 (0.13)	0.006 (0.08)	0.018 (0.13)	0.017 (0.13)	0.017 (0.18)	0.462
Incontinence pad use						
% of patients using incontinence pads	29.4	17.3	20.6	32.8	52.9	<0.0001
Weekly cost of incontinence pads (SD)	KRW 1351 (8465)	KRW 227 (1032)	KRW 566 (1775)	KRW 863 (3014)	KRW 5259 (19320)	<0.0001
Work Productivity and Activity Impairment (WPAI) due to urinary incontinence						
% work time missed (SD)	2.9 (8.1)	0.6 (2.0)	1.4 (4.7)	4.2 (9.6)	7.0 (12.9)	0.001
% regular activity impairment (SD)	29.0 (28.4)	21.2 (24.3)	26.0 (27.4)	31.5 (31.0)	44.8 (26.9)	0.001
Willingness of patients to try medical procedures^a^ to improve urinary symptoms						
% Willing to try	47.5	37.7	41.2	52.0	62.8	<0.0001

^a^Includes sacral neuromodulation, botulinum toxin injections, electrical stimulation, biofeedback training, bladder denervation, detrusor myomectomy, percutaneous tibial nerve stimulation

## Discussion

In this study of Asian patients with OAB, increased UI severity is significantly associated with poorer symptom-specific and general QOL, increased cost of incontinence pads, and a greater interference with work and regular activities. Multivariable analysis conducted demonstrates the significant impact of UI on QOL even after taking into account other important symptoms (ie, urgency, frequency, nocturia).

The results of this study quantified the impact of UI on QOL by using three different QOL instruments. Importantly, based on the MCIDs for the three QOL instruments employed, the transition between UI-severity categories were consistently associated with a clinically meaningful impact to patients. Unlike the results shown by Agarwal (2014) [3] demonstrating UI being the most bothersome symptom, the multivariable regression analysis of KOBIS showed that all symptoms (ie, UI, frequency, urgency and nocturia) have a similar clinically meaningful impact to patients’ QOL, as shown by the coefficients in the multivariable regression model, which exceed the MCID for I-QOL and OAB-q. In contrast, UI did not remain significantly associated with EQ-5D utility in multivariable regression. This is likely due to the poorer sensitivity of generic instruments, such as the EQ-5D, to health dimensions that are affected by the treatment of UI. Compared to incontinence-specific QOL instruments, generic instruments fail to capture the full effect of poor sleep and psycho-social outcomes that are affected by incontinence [35, 36].

Incontinence severity was also identified to be associated with increased weekly cost of incontinence pads, a reduction in hours of work, and increased interference with regular activities. Multivariable analyses adjusted for other OAB symptoms showed that UI was the only significant predictor of pad costs and impact on hours worked, in contrast, the number of urgency episodes per day was the key predictor of interference with daily activities.

In the present study, approximately 50 % of patients were currently or had previously used anti-cholinergic medication. Increasing UI severity was significantly associated with lower current use of anti-cholinergics (*p* < 0.001), and a corresponding higher rate of prior use (*p* = 0.001). This inverse relationship could indicate that severe patients may represent a more difficult to treat group with either lower compliance, or poorer treatment response, which resulted in the lower continued use of anti-cholinergics. The higher doses of anti-cholinergics required by patients with severe UI resulted in greater intolerance due to treatment side effects, such as impaired cognition, constipation, dry mouth, tachycardia, and blurred vision [37]. However, this could not be formally assessed as dosage was not recorded. The study also revealed that only 3.3 % of participants had undergone medical procedures to treat their UI, in contrast, 48 % of patients responded that they would be willing to try these procedures if it could improve their urinary symptoms. This suggests a lack of awareness of treatment options by OAB patients. Recent multi-centered randomized controlled trials [34, 38] have demonstrated the efficacy and safety of botulinum toxin injections for the treatment of OAB symptoms (eg, UI, frequency, urgency, nocturia) in patients inadequately managed with anti-cholinergics. Accordingly, treating clinicians could play a critical role in educating and offering patients effective and well-tolerated alternative treatments.

Consistent with findings from IBIS [14], a trend of greater health service utilisation with increased UI severity was observed, however, this did not achieve statistical significance in the present study. The low rate of prior surgical treatment in the study population may serve to mask the relationship between UI severity and resource use. In addition, patients were recruited through hospital urology clinics where a standard set of diagnostic and monitoring tests are ordered for patients presenting with OAB irrespective of UI severity. Furthermore, it is important to note that the sample size for KOBIS was determined based on power to detect changes in QOL measures. Consequently, the study likely lacked sufficient power to detect changes in secondary outcomes, particularly outcomes with larger variance such as the rate of co-morbidities and resource utilisation.

A potential limitation to this study is the cross-sectional design, which precludes the ability to make definitive causal inferences between UI severity and the outcomes examined. The study is also subjected to recall bias if patients’ ability to recall past events is influenced by the severity of their incontinence. The inclusion of only patients with UI due to idiopathic OAB precludes the applicability of findings in this study to other aetiologies of incontinence (eg, stress incontinence, neurogenic bladder disorders) Lastly, as KOBIS only recruited patients through urology clinics, the lack of patient recruitment from urogynaecologist practices or primary care clinics has the potential to introduce Berkson’s bias. However, the specialist referral system is not widely utilized in Korea, instead, the majority of patients self-refer to specialist clinics, which reduces the likelihood of selection bias related to referral patterns.

## Conclusion

KOBIS is the first study to assess and demonstrate the impact of UI severity on QOL, resource use, and productivity in an Asian population with OAB. Accordingly, treatments that effectively reduce UI will likely improve patients’ QOL and reduce direct costs. The study analyses the collective effect of the four cardinal symptoms of OAB (ie, urinary urgency, frequency, incontinence, and nocturia). These results iterate urinary incontinence as the key contributor to the disease burden of OAB, while also highlighting the importance of treatments that address all aspects of OAB in order to maximise patient outcomes. While traditional treatments such as anti-cholinergic drugs are effective, compliance and use in patients are limited due to their unfavourable tolerability profiles. Therefore, treating clinicians could play a critical role in educating and offering patients effective and well-tolerated treatments options.

### Ethics, consent and permissions

The study was approved by the relevant institutional ethics review boards namely: Samsung Medical Center, Sungkyunkwan University School of Medicine Asan Medical Center, University of Ulsan College of Medicine Cheil General Hospital, Dankoo University College of Medicine Seoul National University Hospital Konkuk University School of Medicine, Konkuk University Medical Center Bucheon St. Mary's Hospital. All participants provided written consent prior to the administration of the questionnaire.

## Additional file

Additional file 1: Table S1. Prevalence of co-morbidities occurring in >5 % of patients. **Table S2.** Patient OAB-related treatment history.
