# Supplementary material for: Impact of overactive bladder on quality of life and resource use: results from Korean Burden of Incontinence Study (KOBIS)
Source: Health Qual Life Outcomes. 2015 Jun 26;13:89. doi: 10.1186/s12955-015-0274-9 (PMC4480453; doi:10.1186/s12955-015-0274-9)
Supplement: Additional file 1: Table S1. — Prevalence of co-morbidities occurring in >5 % of patients. Table S2. Patient OAB-related treatment history. [file 12955_2015_274_MOESM1_ESM.doc]

**Table S**1. Prevalence of co-morbidities occurring in >5% of patients

|  | **All patients** | **Incontinence episode per day** | | | | |
| --- | --- | --- | --- | --- | --- | --- |
|  |  | **0** | **1** | **2-3** | **≥4** | **p-value^a^** |
| N | 625 | 162 | 165 | 177 | 121 |  |
| Childbirth (%) | 34.9 | 29.6 | 33.9 | 35.6 | 42.1 | 0.039 |
| High Blood Pressure (%) | 26.2 | 15.4 | 29.1 | 28.8 | 33.1 | 0.005 |
| Arthritis (%) | 23.8 | 14.2 | 23.6 | 27.7 | 31.4 | 0.027 |
| High Cholesterol (%) | 14.9 | 10.5 | 12.7 | 15.8 | 22.3 | 0.209 |
| Benign Prostatic Hyperplasia (%) | 14.6 | 19.8 | 18.2 | 11.3 | 7.4 | 0.406 |
| Back pain (%) | 13.8 | 8 | 12.7 | 18.6 | 15.7 | 0.056 |
| Diabetes (%) | 11.5 | 8 | 5.5 | 16.9 | 16.5 | 0.017 |
| Urinary tract infections (%) | 10.1 | 11.7 | 4.2 | 13 | 11.6 | 0.995 |
| Osteoporosis (%) | 9.3 | 5.6 | 8.5 | 11.3 | 12.4 | 0.209 |
| Eye disorders (%) | 7.7 | 4.3 | 6.1 | 13.6 | 5.8 | 0.229 |
| Depression (%) | 7.5 | 6.2 | 8.5 | 5.6 | 10.7 | 0.614 |
| Skin infections related to UI (%) | 7.0 | 3.1 | 4.8 | 7.3 | 14.9 | 0.002 |
| Thyroid disease (%) | 6.1 | 6.8 | 5.5 | 6.2 | 5.8 | 0.332 |
| Heart Disease (%) | 5.8 | 3.7 | 6.7 | 5.1 | 8.3 | 0.105 |
| Falls and fractures related to UI (%) | 4.8 | 4.3 | 2.4 | 6.2 | 6.6 | 0.361 |
| Urinary retention (%) | 4.0 | 3.7 | 4.2 | 3.4 | 5 | 0.648 |
| Dehydration (%) | 1.4 | 0.6 | 1.2 | 1.7 | 2.5 | 0.282 |

^a^ Comparison across severity groups conducted using linear or logistic regression for continuous and dichotomous outcomes, respectively. Where appropriate, analysis adjusted for age and gender.

**Table S**2. Patient OAB-related treatment history

|  | **All patients** | **Incontinence episode per day** | | | | |
| --- | --- | --- | --- | --- | --- | --- |
|  |  | **0** | **1** | **2-3** | **≥4** | **p-value** |
| N | 625 | 162 | 165 | 177 | 121 |  |
| Bladder training, n (%) |  |  |  |  |  |  |
| - Currently | 146 (23.4) | 48 (29.6) | 36 (21.8) | 36 (20.3) | 26 (21.5) | 0.109 |
| - In the past | 38 (6.1) | 10 (6.2) | 11 (6.7) | 9 (5.1) | 8 (6.6) | 0.949 |
| - Never | 441 (70.6) | 104 (64.2) | 118 (71.5) | 132 (74.6) | 87 (71.9) | 0.142 |
| Diet modifications, n (%) |  |  |  |  |  |  |
| - Currently | 126 (20.2) | 35 (21.6) | 38 (23) | 30 (16.9) | 23 (19) | 0.276 |
| - In the past | 25 (4) | 9 (5.6) | 9 (5.5) | 5 (2.8) | 2 (1.7) | 0.182 |
| - Never | 474 (75.8) | 118 (72.8) | 118 (71.5) | 142 (80.2) | 96 (79.3) | 0.104 |
| Acupuncture, n (%) |  |  |  |  |  |  |
| - Currently | 7 (1.1) | 5 (3.1) | 1 (0.6) | 1 (0.6) | 0 (0) | 0.028 |
| - In the past | 18 (2.9) | 2 (1.2) | 6 (3.6) | 5 (2.8) | 5 (4.1) | 0.185 |
| - Never | 600 (96) | 155 (95.7) | 158 (95.8) | 171 (96.6) | 116 (95.9) | 0.829 |
| Herbal medication therapy, n (%) |  |  |  |  |  |  |
| - Currently | 13 (2.1) | 3 (1.9) | 5 (3) | 3 (1.7) | 2 (1.7) | 0.725 |
| - In the past | 25 (4) | 6 (3.7) | 6 (3.6) | 8 (4.5) | 5 (4.1) | 0.744 |
| - Never | 587 (93.9) | 153 (94.4) | 154 (93.3) | 166 (93.8) | 114 (94.2) | 0.953 |
| Clean intermittent Catheterisation, n (%) |  |  |  |  |  |  |
| - Currently | 5 (0.8) | 1 (0.6) | 1 (0.6) | 1 (0.6) | 2 (1.7) | 0.416 |
| - In the past | 2 (0.3) | 1 (0.6) | 0 (0) | 0 (0) | 1 (0.8) | 0.907 |
| - Never | 618 (98.9) | 160 (98.8) | 164 (99.4) | 176 (99.4) | 118 (97.5) | 0.452 |
| Indwelling catheter, n (%) |  |  |  |  |  |  |
| - Currently | 0 (0) | 0 (0) | 0 (0) | 0 (0) | 0 (0) | NA |
| - In the past | 4 (0.6) | 2 (1.2) | 1 (0.6) | 0 (0) | 1 (0.8) | 0.442 |
| - Never | 621 (99.4) | 160 (98.8) | 164 (99.4) | 177 (100) | 120 (99.2) | 0.442 |
| Other medical procedures, n (%)* |  |  |  |  |  |  |
| - Currently | 7 (1.1) | 2 (1.2) | 0 (0) | 3 (1.7) | 2 (1.7) | 0.455 |
| - In the past | 14 (2.2) | 5 (3.1) | 2 (1.2) | 1 (0.6) | 6 (5.0) | 0.572 |
| - Never | 604 (96.6) | 155 (95.7) | 163 (98.8) | 173 (97.7) | 113 (93.4) | 0.368 |

*Includes sacral neuromodulation, botulinum toxin injections, electrical stimulation, biofeedback training, bladder denervation, detrusor myomectomy, percutaneous tibial nerve stimulation.
